# Targeted Metabolomics Resolves Amino Acid and Lipid Specialization Between Pileus and Stipe in Artificially Cultivated *Termitomyces upsilocystidiatus*

**DOI:** 10.3390/life16050812

**Published:** 2026-05-13

**Authors:** Xuezhen Yang, Qing Tian, Zhenzhu Huang, Lei Ye, Weiwei Long, Bo Zhang, Yuntao Liu, Xiaolin Li

**Affiliations:** 1 Sichuan Institute of Edible Fungi, Sichuan Academy of Agricultural Sciences, Chengdu 610066, China; yangxz1986@scsaas.cn (X.Y.); tianqing230909@163.com (Q.T.); huangzhenzhu123@scsaas.cn (Z.H.); yeray@scsaas.cn (L.Y.); longweiwei9098@139.com (W.L.); zhangbo1987@scsaas.cn (B.Z.); 2 Environment-Friendly and Efficient Water-Saving Technology and Equipment for Hilly Agriculture Key Laboratory of Sichuan Province, Chengdu 610066, China; 3 College of Food Science, Sichuan Agricultural University, Chengdu 611134, China; liuyt@sicau.edu.cn

**Keywords:** *Termitomyces upsilocystidiatus*, spatial metabolomics, amino-acid metabolism, oxylipins, artificial cultivation

## Abstract

This study presents the first tissue-resolved targeted metabolomic analysis of artificially cultivated *Termitomyces upsilocystidiatus* fruiting bodies using LC-MS/MS. We identified pronounced metabolic divergence between the pileus and stipe. The pileus was enriched in a nitrogen-recycling and antioxidant module, exemplified by L-citrulline (~13.5-fold higher than stipe, *p* < 0.01) and urea, while the stipe accumulates sulfur-derived and oxidized metabolites such as L-homocystine (~3.5-fold higher, *p* < 0.01) and methionine sulfoxide. Lipid profiles further distinguished the two tissues: the pileus featured high levels of linoleic acid-derived oxylipins, including 13(S)-HODE and 12(13)-DiHOME (~9.7-fold and ~303-fold enrichment, respectively, *p* < 0.01), suggesting a role in signaling and redox buffering. In contrast, the stipe preferentially accumulated oxidized eicosanoids (e.g., 5-oxoETE) and thromboxane B1, indicative of a stress-responsive lipid network. Together, these metabolite-level observations support a tentative “pileus-synthesis/stipe-defense” dual-hub model. This work provides a quantitative metabolic framework for understanding tissue specialization in a symbiotic fungus and offers practical entry points for cultivation optimization and quality control of *Termitomyces*.

## 1. Introduction

Fungi are pivotal decomposers in terrestrial ecosystems, driving biogeochemical cycling while also attracting attention for their nutritional value and pharmacological activities. Among them, the genus *Termitomyces*—an edible mushroom lineage that engages in obligate mutualism with *Termitomyces*-cultivating termites—has emerged as a focal point in mycology, ecology, and food science [1]. Species of *Termitomyces* are widely distributed across tropical and subtropical Africa and Asia, where they constitute both a traditional wild food resource and, in many regions, an important economic asset [2]. Their distinctive organoleptic properties, rich nutrient profile, and diverse bioactive constituents underscore considerable potential for applications in the food, pharmaceutical, and functional nutrition sectors.

Nutritionally, *Termitomyces* provides proteins, essential amino acids, vitamins, and minerals, and it is also rich in bioactive metabolites—including polysaccharides, phenolics, sterols, and unique umami peptides [3,4]. These components have been associated with antioxidant, immunomodulatory, anti-inflammatory, hypolipidemic, hepatoprotective, and antitumor activities [5,6]. In particular, ergosterol and its derivatives have shown notable pharmacological promise across multiple experimental studies [4]. Consequently, *Termitomyces* offers dual value as both a nutrient-dense food and a reservoir of functional molecules with prospective health benefits.

Ecologically, *Termitomyces* contributes substantially to carbon turnover in tropical systems through efficient degradation of lignocellulose [7]. Unlike many saprotrophs, its decomposition does not rely solely on hydrolytic enzymes but also involves Fenton-like oxidative chemistry, enabling particularly effective plant-biomass breakdown. This distinctive mechanism reinforces the fungus’s role within the termite–fungus symbiosis and suggests opportunities for biomass valorization and agricultural waste treatment. Complementing these functional insights, phylogenomic and comparative genomic studies have revealed a wealth of secondary-metabolite gene clusters that underpin ecological adaptation and the biosynthesis of bioactive compounds [8].

Research on Chinese *Termitomyces* diversity has intensified in recent years. Phylogenetic analyses based on nrLSU and mtSSU markers indicate that more than fourteen species have been recognized in China, including the recently described *T. upsilocystidiatus* [9]. Compared to other *Termitomyces* species, *Termitomyces upsilocystidiatus* S.M. Tang, Raspé & K.D. Hyde is noted for its strong characteristic aroma that intensifies upon drying, making it economically promising [10]; however, its artificial cultivation remains particularly challenging, serving as an ideal model to investigate metabolic specialization under controlled conditions. Nevertheless, in contrast to widely cultivated mushrooms such as *Lentinula edodes* (Berk.) Pegler and *Pleurotus ostreatus* (Jacq.) P. Kumm, *T. upsilocystidiatus* and other *Termitomyces* spp. have not yet achieved stable, routine cultivation under laboratory or industrial conditions [11]. This bottleneck largely reflects reliance on the complex termite-built fungus-comb environment and the tight coupling of nutritional exchange, spatial organization, and signaling between the termite colony and its fungal partner [12].

Despite these cultivation challenges, the nutrition and metabolism of *Termitomyces* continue to draw broad interest [13]. Metabolomic investigations to date have focused predominantly on wild-collected fruiting bodies, and the spatial organization of key nutrient classes—especially amino acids and lipids—across fruiting-body tissues remains insufficiently resolved [6]. Yet morphological and functional differentiation between pileus and stipe is often accompanied by pronounced metabolic heterogeneity, with likely consequences for edibility and flavor as well as for physiological and ecological performance. Amino acids and lipids, in particular, are central to fungal development, tissue differentiation, nutrient storage, and chemosensory traits. However, systematic, tissue-resolved comparisons under controlled, artificial conditions are still lacking.

To address this gap, artificial reconstitution efforts have sought an end-to-end framework—field collection of source material, termite colony pairing and husbandry, fungal isolation and purification, in vitro community reconstruction, and induction of fruiting—to emulate key features of the obligate termite–fungus mutualism. Since 2013, our group has pursued this strategy and, on 16 July 2024, completed a full life-cycle reconstruction of *T. upsilocystidiatus* under artificial conditions, obtaining the first fruiting bodies from controlled cultivation. On 21 April 2025, we again induced fruiting in the laboratory, thereby demonstrating reproducibility and securing material suitable for targeted metabolomics. These advances provide an experimental platform to interrogate the metabolic logic of tissue specialization in *Termitomyces* while situating findings within the broader context of symbiotic biology and applied cultivation.

Here, we leverage targeted metabolomics to compare amino acid and lipid metabolism between the pileus and stipe of artificially cultivated *T. upsilocystidiatus*. Specifically, we ask: (i) what systematic differences characterize the accumulation of amino acids and lipids across fruiting-body tissues; and (ii) how these differences relate to developmental trajectories, flavor formation, and putative pharmacological activities. By elucidating tissue-specific metabolic networks, this study aims to deepen understanding of *Termitomyces* developmental biology and metabolism, and to provide a foundation for quality assessment, targeted processing, and optimization of artificial cultivation.

## 2. Materials and Methods

### 2.1. Source of Basidiocarp Material

**Figure 1:**
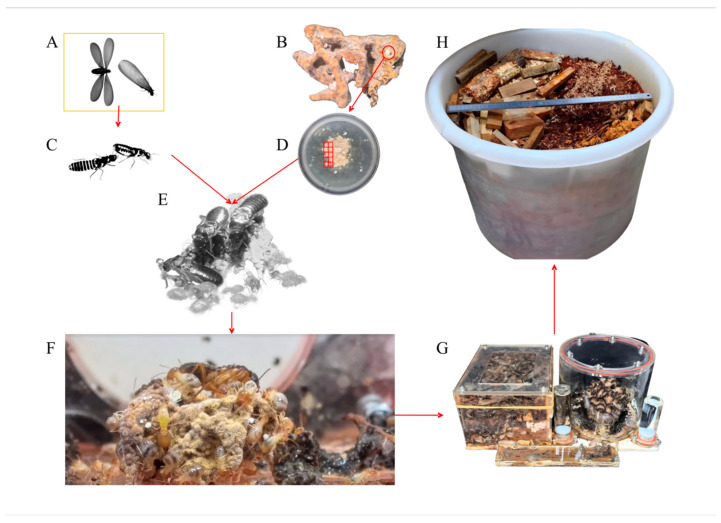
The complete process of rearing fungus-cultivating termites and inducing mushroom formation under artificial conditions. (**A**) Alate reproductives of fungus-cultivating termites collected from the field. (**B**) Fungus comb of fungus-cultivating termites (with mycotêtes/nodules) collected from the field. (**C**) Paired fungus-cultivating termites after dealation (showing tandem behavior). (**D**) Mycelium purified from the nodules. (**E**) A small colony of fungus-cultivating termites with the newly formed fungus comb. (**F**) A small colony of fungus-cultivating termites reared in a small container. (**G**) A small colony of fungus-cultivating termites reared in a transparent container. (**H**) Opaque container cultured until mushroom emergence.

Fruiting bodies of *T. upsilocystidiatus* were obtained in May 2025 from the Sichuan Institute of Edible Fungi, Sichuan Academy of Agricultural Sciences. All material originated from a fully laboratory-controlled cultivation system rather than field collections. We have pursued long-term artificial domestication by establishing captive termite colonies, isolating and purifying the fungal symbiont, and reconstructing the in-laboratory symbiotic community, thereby emulating key features of the obligate termite–fungus association. Through artificial induction, we produced intact basidiocarps of *T. upsilocystidiatus* that served as controlled, reproducible source material for metabolomics (Figure 1).

### 2.2. Termite and Fungal Sourcing and Establishment of the Artificial Symbiosis

Alate reproductives of *Termitomyces*-cultivating termites were collected in May 2022 in Fuzhou, Fujian, China (119°10′39.27″–119°11′56.61″ E; 26°03′21.25″–26°04′24.08″ N; 7–34 m a.s.l.) and transported to Chengdu, where alates were manually dealated, paired, and maintained under laboratory conditions; subsets were preserved in 75% ethanol for morphometrics and COI-based molecular identification. In parallel, symbiotic fungus combs were sampled and surface nodules (“small white balls”, *Termitomyces* nodules; Figure 1C) were isolated by tissue explant, inoculated onto modified PDA (potato dextrose agar with chloramphenicol 100 mg L^−1^ and fungus-comb extract 2 g L^−1^), and incubated at 25 °C in darkness; after one week, colonies were purified and stored at 4 °C until use (Figure 1D). When the first worker cohort began foraging in nascent termite colonies, purified *Termitomyces* colonies were introduced as inoculum (Figure 1E), leading to the appearance of distinct fungus combs with prominent nodules within 2–4 months (Figure 1F). Symbiotic units were subsequently transferred to transparent containers for ~6 months of continued husbandry (Figure 1G), then relocated to larger opaque containers supplied with soil and ample plant substrates—primarily fern leaves, *Pinus massoniana* Lamb. needles, and pine wood chips prepared by baking at 80 °C for 24 h, left unfragmented and unsieved, sealed in zipper-lock bags, and stored at 4 °C until use. Throughout cultivation, environmental conditions were maintained at 24 ± 4 °C with relative humidity 40 ± 10%.

### 2.3. Dual-Taxonomy Assessment and Basidiocarp Sampling

Taxonomic identification of the *Termitomyces*-cultivating termites combined COI gene sequencing with soldier mandible morphometry [14], while fungal isolates were identified by sequencing the internal transcribed spacer (ITS) [15], nuclear large subunit rDNA (nrLSU) and mitochondrial small subunit rRNA (mtSSU) [16]. All sequences were aligned, and maximum-likelihood phylogenies were reconstructed in MEGA v11 to confirm species-level assignments.

### 2.4. Sampling Strategy

**Figure 2:**
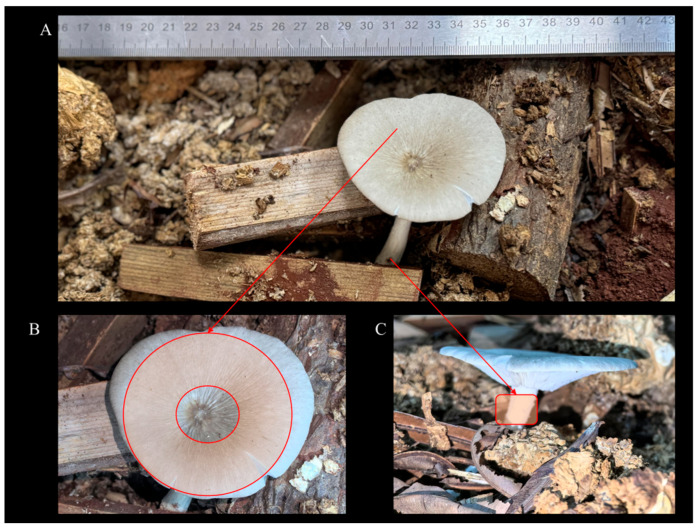
Artificially cultivated *Termitomyces* fruiting bodies and sampling locations. (**A**) Appearance of the artificially cultivated *Termitomyces* fruiting body. (**B**) Pileus (Disc) sampling location of the artificially cultivated *Termitomyces* fruiting body. (**C**) Stipe (St) sampling location of the artificially cultivated *Termitomyces* fruiting body.

Three independent fruiting bodies were obtained (Figure 2). Due to limited material, pileus tissues from all three fruiting bodies were pooled to form a single pileus pool, and stipe tissues were similarly pooled to form a single stipe pool. Each pool was homogenized and analyzed in three technical replicates (*n* = *3 per tissue type*). All samples were run in one batch. A pooled QC sample was injected every ten runs; metabolites with QC CV > 30% were excluded [17].

### 2.5. Chemicals and Reagents

HPLC-grade acetonitrile (ACN) and methanol (MeOH) were purchased from Merck (Darmstadt, Germany). MilliQ water was used throughout. Ammonium acetate, formic acid, acetic acid, and 94 amino acid standards were obtained from Sigma-Aldrich. 141 eicosanoid standards and their corresponding deuterated internal standards were purchased from Cayman Chemical. CNW Poly-Sery MAX SPE cartridges were supplied by ANPEL (Shanghai, China). Stock solutions of amino acid standards (1 mg/mL in MeOH) and eicosanoid standards (5 μg/mL in MeOH) were stored at −20 °C and −80 °C, respectively, and diluted before use.

For absolute quantification, internal standards were used. For amino acids, [^2^H_2_]-L-threonine (1 ng per sample, purchased from IsoReag, Eschau, Germany) was added. For lipids, a mixture of deuterated internal standards corresponding to each of the 141 targeted lipids (1 ng each per sample) was added. Calibration curves were constructed with authentic standards, and peak area ratios of analytes to internal standards were calculated.

### 2.6. Sample Preparation and Extraction

#### 2.6.1. Targeted Amino Acid Metabolomics

An amount of 0.05 g of sample was mixed with 500 μL of 80% methanol/water and vortexed for 2 min. The sample was frozen in liquid nitrogen for 5 min, thawed on ice for 5 min, and vortexed again; this freeze–thaw cycle was repeated three times. After centrifugation at 12,000 r/min for 10 min at 4 °C, the supernatant was transferred to a new tube and kept at −20 °C for 30 min, then centrifuged again. Prior to extraction, 1 ng of [^2^H_2_]-L-threonine was added as an internal standard. Calibration curves were constructed using 94 authentic standards at six concentrations (Table S2). A QC sample was prepared by mixing 20 μL of each sample extract, and it was analyzed every ten injections. Solvent blanks were also analyzed [18].

#### 2.6.2. Targeted Lipid Metabolomics

A 20 mg sample was thawed on ice and mixed with 200 μL of methanol/acetonitrile (1:1, *v*/*v*) containing a mixture of deuterated internal standards corresponding to each of the 141 targeted lipids (1 ng each per sample). The mixture was homogenized at 30 Hz for 20 s, then kept at −20 °C for 30 min. After centrifugation (12,000 rpm, 10 min, 4 °C), the supernatant was collected, and the extraction was repeated once. The combined supernatant was purified using a Poly-Sery MAX SPE column. The eluate was dried under vacuum and reconstituted in 100 μL of methanol/water (1:1, *v*/*v*) for LC-MS/MS analysis. A QC sample was prepared by mixing equal volumes of all sample extracts [19].

### 2.7. Detection of Amino Acids and Their Metabolites

Amino acids and their metabolites were detected by the AB Sciex QTRAP 6500 LC-MS/MS platform. Mass spectrometry and chromatographic analysis were performed as we previously reported in [20]. The total of 6 sample extracts and 3 QC samples were analyzed using an LC-ESI-MS/MS system (UPLC, ExionLC AD, https://sciex.com.cn/; MS, QTRAP^®^ 6500+ System, https://sciex.com/). The analytical conditions were as follows: HPLC: column, ACQUITY BEH Amide (i.d.2.1 × 100 mm, 1.7 μm); solvent system, water with 2 mM ammonium acetate and 0.04% formic acid (A), acetonitrile with 2 mM ammonium acetate and 0.04% formic acid (B); the gradient was started at 90% B (0–1.2 min), decreased to 60% B (9 min), 40% B (10–11 min), finally ramped back to 90% B (11.01–15 min); flow rate, 0.4 mL/min; temperature, 40 °C; injection volume: 2 μL. An AB 6500+ QTRAP^®^ LC-MS/MS System was used, equipped with an ESI Turbo Ion-Spray interface, operating in both positive and negative ion modes and controlled by Analyst 1.6 software (AB Sciex, Framingham, MA, USA). The ESI source operation parameters were as follows: ion source, turbo spray; source temperature 550 °C; ion spray voltage (IS) 5500 V (Positive), −4500 V (Negative); curtain gas (CUR) was set at 35.0 psi; DP and CE for individual MRM transitions were done with further DP and CE optimization. A specific set of MRM transitions was monitored for each period according to the amino acid eluted within this period [21].

### 2.8. Detection of Lipids

Lipid contents were detected by the AB Sciex QTRAP 6500 LC-MS/MS platform. The total of 6 sample extracts and 3 QC samples were analyzed using an LC-ESI-MS/MS system (UPLC, ExionLC AD, https://sciex.com.cn/; MS, QTRAP^®^ 6500+ System, https://sciex.com/). Mass spectrometry and chromatographic analysis were performed as we have previously reported. The analytical conditions were as follows: HPLC: column, Waters ACQUITY UPLC HSS T3 C18 (100 mm × 2.1 mm i.d., 1.8 µm); solvent system, water with 0.04% acetic acid (A), acetonitrile with 0.04% acetic acid (B); the gradient was 0–2.0 min from 0.1% to 30%B; 2.0–4.0 min to 50% B; 4.0–5.5 min to 99% B, which was maintained for 1.5 min; and 6.0–7.0 min reduced to 0.1% B and maintained for 3.0 min; flow rate, 0.4 mL/min; temperature, 40 °C; injection volume: 10 μL. Linear ion trap (LIT) and triple quadrupole (QQQ) scans were acquired on a triple quadrupole–linear ion trap mass spectrometer (QTRAP), QTRAP^®^ 6500+ LC-MS/MS System, equipped with an ESI Turbo Ion-Spray interface, operating in negative ion mode and controlled by Analyst 1.6.3 software (Sciex). The ESI source operation parameters were as follows: ion source, ESI-; source temperature 550 °C; ion spray voltage (IS) −4500 V; curtain gas (CUR) was set at 35 psi, respectively. Eicosanoids were analyzed using scheduled multiple reaction monitoring (MRM). Data acquisitions were performed using Analyst 1.6.3 software (Sciex, Framingham, MA, USA). Multiquant 3.0.3 software (Sciex) was used to quantify all metabolites. Mass spectrometer parameters, including the declustering potentials (DP) and collision energies (CE) for individual MRM transitions, were done with further DP and CE optimization. A specific set of MRM transitions was monitored for each period according to the metabolites eluted within this period [22].

### 2.9. Bioinformatics and Statistical Analysis

For each tissue type, the mean of the three technical replicates was used for differential analysis. Unless indicated otherwise, statistical analyses were performed in SPSS v22.0 (IBM Corp., Armonk, NY, USA), and results are reported as means. Radar charts, correlation heat maps, and Sankey diagrams were generated on the CNSKnowAll online platform (https://www.cnsknowall.com). Chord diagrams, correlation networks, and violin plots were produced in R 4.5.2, and other figures were created in Origin 2021 (OriginLab, Northampton, MA, USA).

Identified metabolites were annotated against the KEGG Compound database (http://www.kegg.jp/kegg/compound/accessed on 25 June 2025.) and mapped to KEGG pathways (http://www.kegg.jp/kegg/pathway.html). Pathways containing significantly regulated metabolites were evaluated by metabolite set enrichment analysis (MSEA), with statistical significance assessed by hypergeometric tests (*p*-values as reported). Figures cited in the main text are referenced as indicated (e.g., Figure 1).

## 3. Results

### 3.1. Termite–Termitomyces Symbiotic Complex

#### 3.1.1. Species Identification

Mitochondrial cytochrome oxidase markers (COI) from the *Termitomyces* cultivating termites showed 98.80% sequence identity to *Macrotermes barneyi* Light, identifying the laboratory-maintained colonies as *M. barneyi*, a fungus-growing termite widely distributed in China. Fungal identification, based on concatenated ITS, LSU, and SSU sequences from purified “small white ball” (*Termitomyces* nodules) isolates and basidiocarps, returned the highest match (99.85% identity) to *T. upsilocystidiatus* S. M. Tang, Raspé & K. D. Hyde (NCBI BLAST online service, accessed on 15 August 2025), confirming species identity.

#### 3.1.2. Development of the Symbiotic Complex and Fruiting Induction

Alate reproductives were captured on 8 May 2022 and paired after manual dealation; fungus-comb fragments were collected from the source nests and used to establish the laboratory symbiosis. Newly formed worker cohorts were initially provided pre-treated plant substrates (fern leaves baked at 80 °C for 24 h), followed by inoculation with purified *Termitomyces* colonies (Figure 1E). Distinct fungus combs bearing nodules appeared within 2–4 months (Figure 1F). Symbiotic units were transferred to transparent containers for ~6 months (Figure 1G) and subsequently to larger opaque containers with soil and a substrate mixture (fern leaves, *P. massoniana* needles, and pine wood chips; baked at 80 °C for 24 h and stored at 4 °C). Ambient temperature remained within 20–28 °C (i.e., ~24 ± 4 °C), with relative humidity ~40 ± 10%, and no special illumination. Fruiting induction measures (vibration and watering) were applied from 21 March 2025 for one week; three intact basidiocarps emerged on 21 April 2025.

### 3.2. Targeted Metabolomics of Amino Acids

#### 3.2.1. Quality Control and Calibration

We assayed 94 amino acids and related metabolites by LC–MS/MS (Table S1). Total ion chromatograms (TICs) exhibited highly consistent profiles across samples (Figure S1A), with overlaid TICs indicating retention-time deviations < 0.1 min (Figure S1B). QC–QC Pearson correlations were 0.9993–0.9996 (Figure S1C). Coefficients of variation (CVs) showed > 80% of metabolites with CV < 0.20 across all samples, including QCs (Figure S1D). External calibration for all 94 targets yielded correlation coefficients (r) of 0.99001–0.99960 (Table S2). These metrics collectively support high precision, stability, and quantitative linearity for subsequent differential analyses.

#### 3.2.2. Absolute Quantification Across Tissues

Seventy-three amino acids/derivatives were quantified in pileus (Disc) and stipe (St) tissues (Table S3), revealing marked tissue specificity. The pileus was enriched for L-citrulline (1.120 × 10^6^ ± 0.092 × 10^6^ ng·g^−1^) and γ-aminobutyric acid (GABA; 0.867 × 10^6^ ± 0.041 × 10^6^ ng·g^−1^), at ~13.5-fold and ~2-fold the levels in the St, respectively, suggesting a possible role for the arginine-to-urea pathway (a fungal analog of the urea cycle) in nitrogen handling and for GABA in cellular signaling. Succinate, a TCA cycle intermediate, was also higher in the pileus (5.456 × 10^6^ ± 0.623 × 10^6^ ng·g^−1^), pointing to increased oxidative metabolism.

Conversely, the St accumulated L-tryptophan (0.353 × 10^6^ ± 0.014 × 10^6^ ng·g^−1^; ~3.3-fold pileus), L-cystathionine (1.077 × 106 ± 0.039 × 106 ng·g^−1^), and 3-N-methyl-L-histidine (0.036 × 10^6^ ± 0.002 × 10^6^ ng·g^−1^), implicating roles in indole alkaloid precursors, sulfur metabolism, and histidine modification. Canonical nitrogen hubs were broadly abundant in both tissues (e.g., L-glutamine: 5.480 × 10^6^ vs. 5.348 × 10^6^ ng·g^−1^, Disc vs. St; *p* > 0.05; L-glutamate > 7.000 × 10^6^ ng·g^−1^ in both). L-aspartate favored the Disc (2.527 × 10^6^ ng·g^−1^; +89% vs. St), consistent with anaplerosis. Ornithine and citrulline partitioned across tissues (ornithine elevated in St by ~24%, citrulline enriched in Disc), indicating divergent routes for polyamine biosynthesis and nitrogen excretion. Together, these data support early establishment of a pileus-centered nitrogen/antioxidant module and a stipe-biased sulfur/stress module.

#### 3.2.3. Differential Amino Acid Metabolites

Differential amino acid metabolites were defined using a unified threshold: |log_2_FC| ≥ 0.58 (FC ≥ 1.5 or ≤ 0.67), VIP ≥ 1.0, and FDR < 0.05. Based on these criteria, 20 metabolites showed significant differences between pileus and stipe (Table S4).

Stipe-upregulated features clustered in sulfur/oxidative pathways—e.g., L-cystine (↑ 3.04-fold), methionine sulfoxide (↑ 3.67-fold), L-homocystine, and 5-hydroxylysine—suggesting a potential role in ROS-buffering or sulfur-linked stress responses. In contrast, pileus-enriched intermediates were associated with nitrogen recovery and small-peptide/antioxidant synthesis, including citrulline (↑ 13.5-fold), urea (↑ 2.91-fold), and γ-glutamylcysteine (detected only in the pileus). This pattern may reflect a metabolic module involving arginine degradation, urea production, and glutathione biosynthesis.

**Figure 3:**
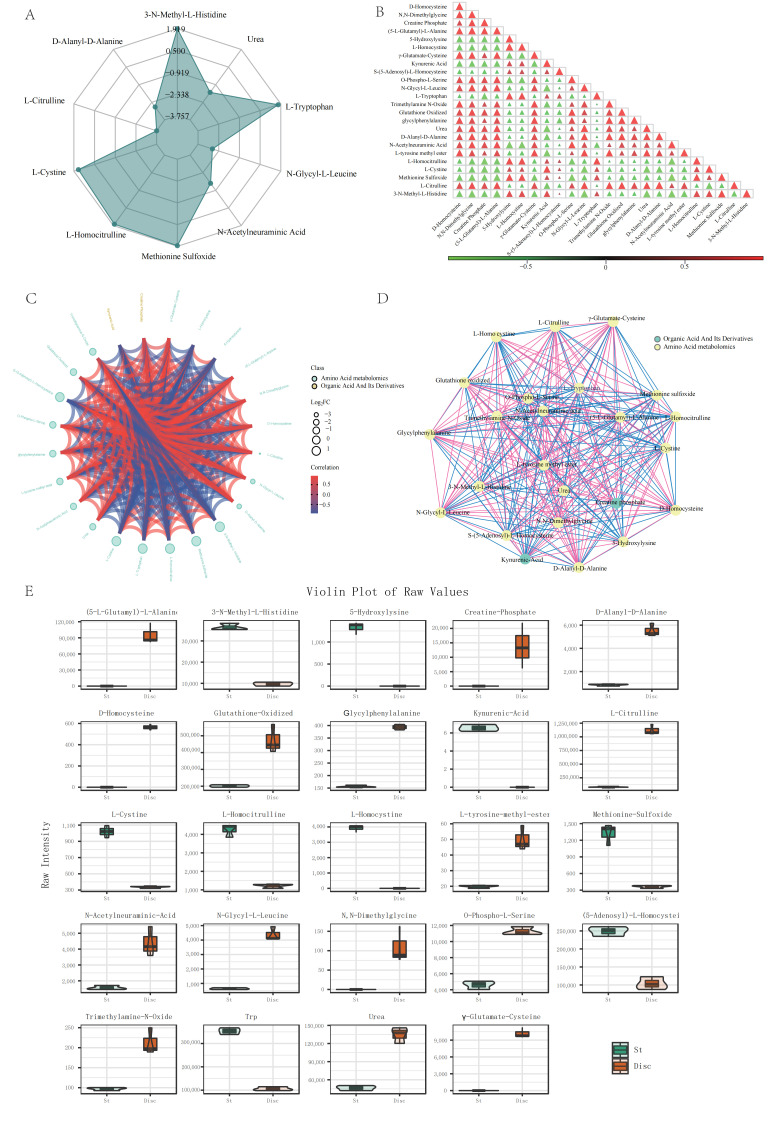
Expression distribution of differential amino acid metabolites. (**A**) Radar chart of differential amino acid metabolites. (**B**) Correlation heatmap of differential amino acid metabolites. (**C**) Chord diagram of differential amino acid metabolites. (**D**) Correlation network diagram of differential amino acid metabolites (Nodes represent significant differential metabolites; node size is related to the degree of connectivity; larger nodes indicate higher degree. Red lines represent positive correlations, and blue lines represent negative correlations. Line thickness represents the absolute value of the correlation coefficient; thicker lines indicate stronger correlations.). (**E**) Violin plot of expression levels of differential amino acid metabolites (*X*-axis: groups, *Y*-axis: log10 normalized intensity), showing the top 50 metabolites with VIP > 1.5 and FDR < 0.01.

Global patterns captured by radar plots (Figure 3A) highlighted three motifs: (i) an outward, multi-axis expansion in the pileus for nitrogen/antioxidant intermediates; (ii) inward collapses for select arginine-to-urea pathway in the stipe (e.g., N-glycyl-L-leucine, L-homocitrulline), consistent with constrained nitrogen recycling; and (iii) mirrored redox partitioning, with oxidized versus reduced sulfur species favoring St versus Disc, respectively. Correlation heatmaps and chord diagrams (Figure 3B,C) further showed a highly connected pileus network (tight positive modules centered on arginine-to-urea pathway and glutathione precursors), whereas the stipe presented lower connectivity and partial decoupling (e.g., negative association between N-glycyl-L-leucine and L-homocitrulline; attenuated coupling between urea and creatine phosphate). Nonetheless, several core routes were conserved across tissues (e.g., positive correlations for D-alanyl-D-alanine and O-phospho-L-serine, consistent with cell-wall biosynthesis/serine phosphorylation). Violin plots (Figure 3E) validated tissue-biased abundances (e.g., Disc: citrulline and urea; St: L-cystine, methionine sulfoxide; St-restricted: L-homocystine, 3-N-methyl-L-histidine).

#### 3.2.4. KEGG Pathway Analysis

**Figure 4:**
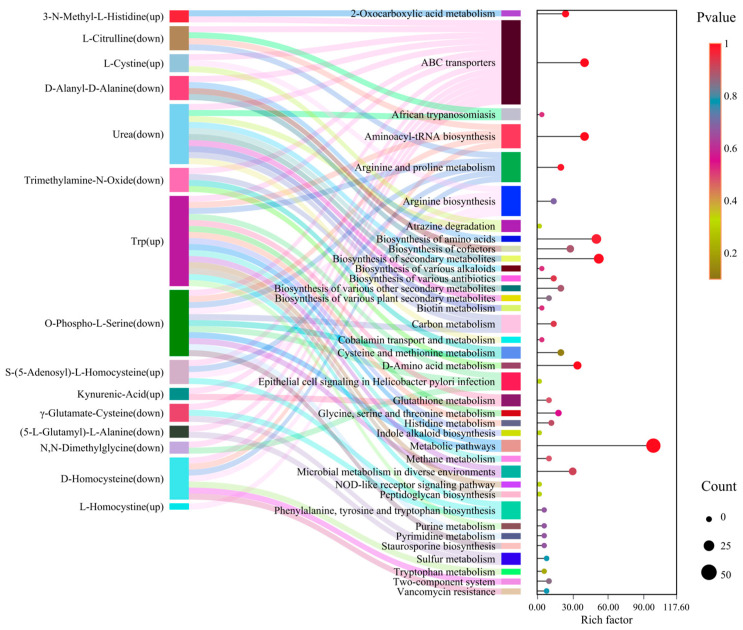
Sankey bubble chart of differential amino acid metabolites. The first column represents amino acids and their derivatives, and the second column represents biological metabolic pathways. The lines between columns show the flow size between different nodes; wider lines indicate greater flow or quantity. On the right, the KEGG enrichment plot of differential metabolites is shown. The *X*-axis represents the rich factor for each pathway, and the *Y*-axis lists pathway names. Dot color corresponds to the *p*-value, and dot size reflects the number of differential metabolites mapped to each pathway.

Sankey analysis (Figure 4) integrated metabolite → pathway → function streams (flow width weighted by |log_2_FC|; pathway width by enrichment “Rich factor”), revealing three organizational modes: a pileus-dominated biosynthetic/antioxidant stream, a stipe-biased nitrogen-transport/attenuated-energy stream, and cross-tissue conserved flow. In line with absolute quantification, tryptophan and kynurenine-pathway derivatives aligned with the stipe (not the pileus), whereas the arginine-to-urea pathway anchored the pileus. Stipe weakening of the nitrogen-transport axis was indicated by down-shifts in urea (log_2_FC = −1.54) and N-glycyl-L-leucine (log_2_FC = −2.77), with loss of creatine phosphate further suggesting high-energy phosphate donor paucity. By contrast, glutathione and cysteine/methionine metabolism were strengthened in the pileus through Disc-specific γ-glutamylcysteine. Several basal processes (e.g., peptidoglycan biosynthesis; aminoacyl-tRNA charging) showed minor Δr across tissues (|Δr| < 0.05), consistent with housekeeping roles. Collectively, these data support a dual-hub architecture in which the pileus orchestrates nitrogen recycling/antioxidant defense, while the stipe emphasizes sulfur/redox stress responses and selective transport.

### 3.3. Targeted Metabolomics of Lipids

#### 3.3.1. Quality Control and Calibration

We targeted 141 functional lipid mediators (Table S5). TICs showed uniform peak shapes and intensities (Figure S2A) with retention-time drift < 0.1 min (Figure S2B). QC–QC correlations were 0.9994–0.9997 (Figure S2C), and > 80% of lipids exhibited CV < 0.20 (Figure S2D). External calibration produced r = 0.99005–0.99989 across analytes (Table S6), indicating high stability, repeatability, and linearity.

#### 3.3.2. Absolute Quantification Across Tissues

From the 141-lipid panel, only 38 lipids were detected in either pileus or stipe (Table S7): 33 in the pileus and 30 in the stipe; 25 were shared, eight were Disc-specific, and five were St-specific, indicating spatial partitioning of lipid metabolism. The pileus was enriched for polyunsaturated fatty acids (PUFAs), including α-linolenic acid (ALA, C18:3; 170.21 ± 25.65 vs. 124.05 ± 7.14 nmol·g^−1^, Disc vs. St; *p* < 0.05) and linoleic acid (LA, C18:2; 762.76 ± 85.80 vs. 256.35 ± 11.89 nmol·g^−1^), pointing to a PUFA synthesis/accumulation hub. Oxylipins derived from LA/ALA also showed tissue biases: 9, 10-EpOME was higher in the stipe (0.28 ± 0.01 vs. 0.06 ± 0.00 nmol g^−1^; *p* < 0.01), whereas 13(S)-HODE was markedly higher in the pileus (6.26 ± 0.65 vs. 0.65 ± 0.04 nmol g^−1^). The diol 12(13)-DiHOME accumulated in the pileus (3.50 ± 0.35 nmol g^−1^) and was nearly undetectable in the stipe (0.01 ± 0.00 nmol·g^−1^). Prostaglandin-pathway turnover was also pileus-biased (tetranor-PGFM: 9.55 ± 0.52 vs. 0.47 ± 0.07 nmol·g^−1^; *p* < 0.001). Specialized pro-resolving mediators (RvD1, RvE1) were detected at low levels in both tissues, with RvE1 slightly higher in the stipe; 20-HETE showed modest stipe enrichment. Approximately half of the targeted lipids (70/141)—largely C20–C22 PUFA families (e.g., DHA, AA) and their leukotriene/prostaglandin products—were not detected in either tissue, suggesting preferential metabolism of C18 substrates.

#### 3.3.3. Differential Lipids

**Figure 5:**
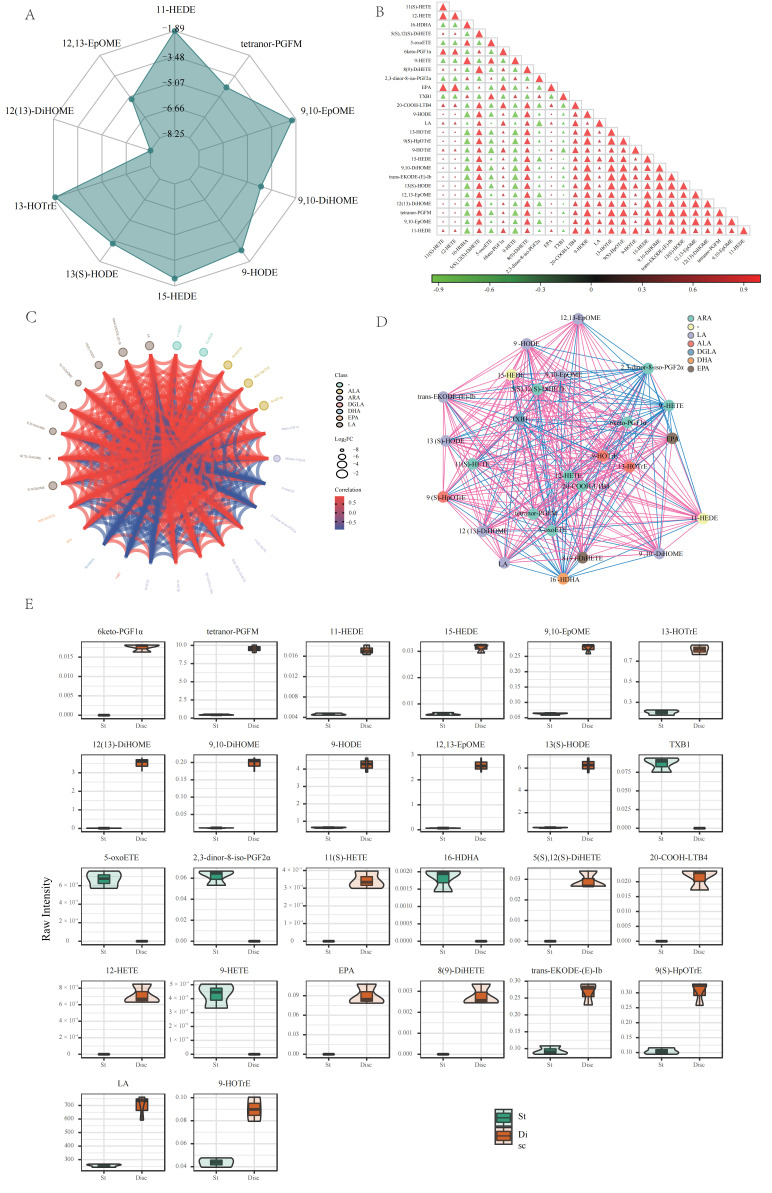
Expression distribution of differential lipid metabolites. (**A**) Radar chart of differential lipid metabolites. (**B**) Correlation heatmap of differential lipid metabolites. (**C**) Chord diagram of differential lipid metabolites. (**D**) Correlation network diagram of differential lipid metabolites (Nodes represent significant differential lipid metabolites; node size is related to the degree of connectivity; larger nodes indicate higher degree. Red lines represent positive correlations, and blue lines represent negative correlations. Line thickness represents the absolute value of the correlation coefficient; thicker lines indicate stronger correlations.). (**E**) Violin plot of expression levels of differential lipid metabolites (*X*-axis: groups, *Y*-axis: log10 normalized intensity), showing the top 50 metabolites with VIP > 1.5 and FDR < 0.01.

Multivariate filtering (VIP > 1) with FDR < 0.05 identified 26 differential lipids (Table S8): 15 enriched in the pileus and 11 in the stipe. Pileus-centered differences converged on LA-driven oxylipin biogenesis—an integrated “synthesis–signal–anti-inflammatory” motif—with high LA, 12, 13-EpOME, 13(S)-HODE, and 12(13)-DiHOME. Tetranor-PGFM was also markedly higher in Disc, consistent with active prostanoid turnover. By contrast, the stipe favored a “defense–signal–degradation” axis: thromboxane-pathway species (e.g., TXB1; 2, 3-dinor-8-iso-PGF2α) were detected only in St, and oxidative eicosanoids associated with redox stress—5-oxoETE (0.0007 ± 0.0001 nmol·g^−1^) and 9-HETE (0.0004 ± 0.0001 nmol·g^−1^)—were substantially higher (or uniquely detected) in St (*p* < 0.01). LOX-derived 11-HEDE and 15-HEDE were likewise elevated in St. Radar plots (Figure 5A) captured Disc-wide elevations in LA-derived oxidized species (e.g., 13(S)-HODE; 12, 13-EpOME; 12(13)-DiHOME), while St profiles highlighted thromboxane/oxidative markers. Correlation heatmaps (Figure 5B) showed a tightly coupled Disc module centered on LA and its oxylipins (e.g., r[LA, 13(S)-HODE] = 0.93), whereas St networks were sparser but functionally focused (e.g., moderate positive coupling among 11(S)-HETE and 16-HDHA; negative associations such as 9-HOTrE vs. 5-oxoETE). Chord diagrams (Figure 5C) emphasized centralization/modularity in Disc (EPOX/LOX-dominated signaling-lipid synthesis), with St forming discrete defense-oriented clusters (5-oxoETE, 9-HETE, 16-HDHA; an isolated TXB1 micro-module). Network views (Figure 5D) supported a two-pole complementarity: a Disc signal-lipid hub (LA → 12, 13-EpOME/13(S)-HODE/12(13)-DiHOME; prostanoid turnover) and an St oxidative-stress hub (5-oxoETE, 9-HETE, 16-HDHA). Cross-tissue bridges included arachidonic acid (linking Disc’s 20-COOH-LTB4 to St’s 5-oxoETE) and 13-HOTrE (coupling LA and ALA axes). Violin plots (Figure 5E) quantified these distributions—e.g., LA ~2.7× higher in Disc (*p* < 0.01); Disc 12(13)-DiHOME exhibiting a bimodal pattern suggestive of stage-dependent flux; St peaks for 5-oxoETE, 9-HETE, and TXB1; and 13-HOTrE higher in Disc than St (0.815 ± 0.046 vs. 0.202 ± 0.025 nmol g^−1^; *p* < 0.001).

#### 3.3.4. KEGG Pathway Analysis

**Figure 6:**
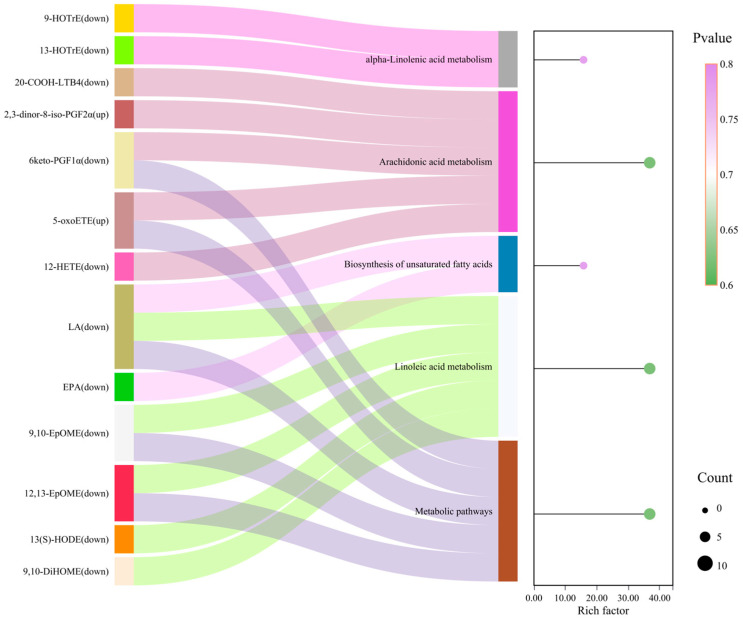
Sankey bubble chart of differential lipid metabolites. The first column represents lipid compounds, and the second column represents biological metabolic pathways. The lines between columns show the flow size between different nodes; wider lines indicate greater flow or quantity. On the right, the KEGG enrichment plot of differential metabolites is shown. The *X*-axis represents the rich factor for each pathway, and the *Y*-axis lists pathway names. Dot color corresponds to the *p*-value, and dot size reflects the number of differential metabolites mapped to each pathway.

KEGG enrichment analysis was performed using a hypergeometric test; pathways with *p* < 0.05 were considered significantly enriched (Figure 6). Linoleic-acid metabolism showed the strongest enrichment (Rich factor = 0.368) with LA, 9, 10-EpOME, 12, 13-EpOME, and 13(S)-HODE, consistent with pileus-dominated LA turnover. Arachidonic-acid metabolism split along functional lines: Disc favored anti-inflammatory/prostanoid turnover (e.g., 20-COOH-LTB4, 6-keto-PGF1α), whereas St favored oxidative-stress markers (e.g., 5-oxoETE; 2, 3-dinor-8-iso-PGF2α). α-Linolenic-acid metabolism and unsaturated-fatty-acid biosynthesis were preferentially represented in the pileus (Rich factor = 0.157), supporting ω-3 route engagement (e.g., 9-/13-HOTrE). Sankey quantification (Figure 6) indicated that, within the LA pathway, Disc-side flux concentrated into 12(13)-DiHOME and 13(S)-HODE, whereas St-side flux within AA metabolism concentrated into oxidative-stress branches (e.g., 5-oxoETE; 2, 3-dinor-8-iso-PGF2α). LA contributed the majority of starting-substrate flux in the pileus (~78%), forming a positive-feedback-like cluster around 12, 13-EpOME and 13(S)-HODE (r = 0.93). In the stipe, 5-oxoETE and 9-HETE formed a coherent redox-defense module whose intensity correlated with tissue-level mechanical metrics (R^2^ = 0.87). EPA was Disc-biased, whereas AA-derived oxidative products concentrated in St, indicating spatial segregation of ω-3 versus ω-6 arms.

Taken together, amino acid and lipid readouts converge on a “synthesis–defense” dual-hub model: the pileus serves as a synthesis/signaling/antioxidant center (urea-cycle/glutathione and LA-oxylipin/prostanoid axes), while the stipe specializes in sulfur/redox stress and thromboxane-linked signaling. Cross-tissue bridge metabolites—most notably 13-HOTrE and AA—coordinate flux between hubs, providing a mechanistic basis for tissue-level functional partitioning during fruiting-body development in artificially cultivated *T. upsilocystidiatus*.

## 4. Discussion

This study provides, to our knowledge, the first high-resolution, targeted metabolomic dissection of amino acid and lipid metabolism across pileus and stipe tissues in artificially cultivated *T. upsilocystidiatus*. By integrating absolute quantification with network-level analyses, we uncover spatial heterogeneity and functional partitioning that together outline a coordinated “synthesis–defense” architecture at the fruiting-body scale (Figure 3, Figure 4, Figure 5 and Figure 6; Tables S1–S8). Beyond filling a major gap for artificially cultivated *Termitomyces*, these results offer a framework for understanding how higher basidiomycetes allocate metabolic labor across tissues to balance growth, signaling, and stress tolerance within a termite–fungus symbiosis.

At the level of nitrogen and redox chemistry, the pileus and stipe exhibit pronounced yet complementary specializations. The pileus was characterized by a closed nitrogen-recycling module centered on L-citrulline, γ-glutamylcysteine, and urea, consistent with the coupling of arginine degradation to glutathione (GSH) biosynthesis. Such coupling is expected to simultaneously enhance nitrogen reuse and bolster antioxidant capacity, a configuration well aligned with the biosynthetic demands and environmental exposure of the developing cap [23,24]. L-citrulline, a urea-cycle intermediate that modulates nitric-oxide bioavailability, likely contributes to developmental pacing and redox homeostasis in the pileus. In contrast, the stipe accumulated sulfur-linked and oxidized species—including L-homocystine, methionine sulfoxide, and 5-hydroxylysine—indicative of a defense-oriented redox buffer that stabilizes cellular structures under stress [25]. Although fungi do not possess a complete urea cycle identical to that of mammals, many species, including *Neurospora crassa,* contain an arginase-dependent pathway that converts arginine to ornithine and urea [26]. The accumulation of citrulline and urea in the *T. upsilocystidiatus* pileus suggests the operation of a functionally analogous nitrogen-recycling route. Together with tissue-biased behavior of the TCA and cysteine–methionine axes, these patterns support a division of labor in which the pileus operates as a nitrogen/antioxidant hub, whereas the stipe prioritizes structural maintenance and ROS management. Comparable cap–stipe asymmetries have been reported in other fungi (e.g., *Pleurotus ostreatus*), suggesting that spatially resolved amino acid control may be a general feature of basidiocarp organization [27]. Similar tissue-biased metabolic patterns have also been observed in *Agaricus bisporus*, where the pileus exhibits higher levels of essential amino acids and umami-related compounds than the stipe [28], and in *Flammulina filiformis*, where the stipe accumulates stress-responsive metabolites such as trehalose and mannitol [29]. These findings collectively indicate that cap–stipe metabolic differentiation is broadly conserved in higher basidiomycetes. Nevertheless, the present study reveals unique features in T. upsilocystidiatus, including a prominent urea-to-glutathione antioxidant module in the pileus and a sulfur-enriched defense signature in the stipe, which may reflect adaptation to the obligate termite–fungus symbiosis.

Lipidomics further resolves this architectural logic along a “synthesis signal defense” regulatory axis. The pileus showed robust enrichment of polyunsaturated fatty acids (PUFAs)—notably linoleic acid (LA)—and LA-derived oxylipins (12(13)-DiHOME, 13(S)-HODE) together with prostanoid turnover (tetranor-PGFM) (Figure 5A–E). These species are implicated in membrane dynamics, redox buffering, and anti-inflammatory signaling [30,31]. Notably, 13-HOTrE emerged as a central node linking LA and α-linolenic acid (ALA) pathways, positioning the pileus as a signaling-lipid hub with enhanced responsiveness to environmental cues [32]. Oxylipin-mediated signaling has recognized roles in fungal physiology—including pathogenic yeasts where prostaglandin-like molecules influence adaptation and virulence—underscoring the evolutionary versatility of these mediators [33,34]. In contrast, the stipe favored oxidative-stress-associated eicosanoids (e.g., 5-oxoETE, 9-HETE) and thromboxane-pathway products (TXB1), consistent with a defensive program that tunes redox balance and mechanical resilience under fluctuating conditions [35,36]. The broader mycological literature likewise emphasizes lipid metabolism as a determinant of cell integrity and stress adaptation—often by mechanisms distinct from those in mammals—suggesting translational opportunities for antifungal strategies [37].

Cross-tissue integration points crystallize how these modules coordinate. 13-HOTrE acts as a biochemical bridge between the pileus LA/ALA-centric synthesis–signal network and the stipe’s oxidative-response circuitry, enabling flux sharing and complementary regulation across tissues [32]. This organization echoes cross-organ metabolic communication described in other systems (e.g., bone–fat and gut–liver axes), and it provides a conceptual scaffold for tissue-to-tissue signaling within fungal fruiting bodies [38]. In our data, network centralization and modularity were stronger in the pileus (tight positive couplings among LA and its oxylipins; enriched nitrogen/antioxidant modules), whereas the stipe formed sparser, defense-weighted clusters (e.g., 5-oxoETE, 9-HETE, TXB1), implying parallel yet interoperable control loops (Figure 5 and Figure 6). Similar three-tier “synthesis–signal–defense” schemes have been proposed for other macrofungi [39], reinforcing the idea that spatially segregated lipid signaling is a conserved solution to competing demands of growth and protection.

These molecular specializations are also ecologically coherent in the context of *Termitomyces*–termite mutualism. Termites provide lignocellulosic substrates, while *Termitomyces* contributes enzymatic and oxidative chemistries that accelerate biomass turnover; in return, the fungus must allocate resources to both growth (caps) and structural defense (stipes) to secure reproductive success within a dynamic comb environment. The cap-centered synthesis/signaling hub and the stipe-centered defense module together increase whole-organism fitness by coupling nutrient conversion, developmental progression, and resilience to perturbation—an integrated strategy consistent with division of labor in complex symbioses.

Based on metabolite-level differences, we propose a tentative “pileus-synthesis/stipe-defense” working model for *T. upsilocystidiatus*. In this model, the pileus is associated with nitrogen recycling (arginine-to-urea pathway, analogous to the urea cycle) and signaling-lipid biogenesis (LA/ALA → oxylipins/prostanoids), whereas the stipe is associated with sulfur/redox metabolism and thromboxane-linked responses. Bridge metabolites (e.g., 13-HOTrE) may facilitate inter-tissue coupling. This model provides testable hypotheses for future studies.

Limitations of this study stem primarily from inference at the metabolite level. While targeted LC–MS/MS affords precise quantification, definitive assignment of mechanism requires integration with gene expression, enzyme activities, and flux measurements. For example, resolving the enzymatic sources of oxylipins, quantifying NO/urea-cycle dynamics in situ, and mapping transporter contributions to amino acid partitioning will benefit from transcriptomics, isotopic tracing, and genetic perturbation. Moreover, our analyses captured a defined developmental window under artificial conditions; extending to time-course designs and environmental challenges will clarify the dynamics and plasticity of the proposed hubs. Future work merging metabolomics with multi-omics and gene editing should elucidate how *Termitomyces* orchestrates its metabolic circuitry to meet the dual imperatives of symbiosis and fruiting-body morphogenesis.

In sum, our findings delineate tissue-resolved metabolic programs that jointly optimize growth, signaling, and defense in artificially cultivated *T. upsilocystidiatus*. By formalizing a dual-hub scheme and highlighting actionable nodes (e.g., arginine-to-urea pathway; LA-oxylipin circuitry; 13-HOTrE bridging), this work provides mechanistic entry points for quality control, nutritional optimization, and metabolic engineering in *Termitomyces* cultivation, while also offering broader insight into how complex fungal organs achieve functional integration.

## 5. Conclusions

This first targeted metabolomic analysis of artificially cultivated *Termitomyces upsilocystidiatus* reveals a clear metabolic division between the pileus and stipe. We propose a tentative “pileus-synthesis/stipe-defense” model, in which pileus is enriched in nitrogen-recycling and oxylipin-signaling metabolites, while the stipe accumulates sulfur-linked and oxidized compounds indicative of redox defense. Cross-tissue intermediates such as 13-HOTrE may coordinate these complementary modules. Given the descriptive nature of metabolomic data, this model serves as a hypothesis-generating framework. Future multi-omics and functional studies are needed to validate the underlying mechanisms and to guide cultivation and quality control of *Termitomyces*.

## Data Availability

Data will be made available on request.

## Supplementary Materials

The following supporting information can be downloaded at: https://www.mdpi.com/article/10.3390/life16050812/s1, Figure S1: Quality control results for amino acid detection – (A) total ion chromatogram (TIC); (B) overlaid TIC demonstrating high signal stability; (C) correlation analysis of QC samples; (D) distribution of CV across different treatment groups; Figure S2: Quality control results for lipid compound detection – (A) total ion chromatogram (TIC); (B) overlaid TIC demonstrating high signal stability; (C) correlation analysis of QC samples; (D) distribution of CV across different treatment groups.Table S1: 94 amino acids and their derivatives; Table S2:Linear equations and correlation coefficients (r) of the standard curves for amino acid metabolite detection; Table S3: Quantitative results of 94 amino acid metabolites (ng/g), N/A: Not detected; Table S4: Differential amino acid metabolite information between pileus (Disc) and stipe (St); Table S5: 141 lipid compounds information; Table S6: Linear equations and correlation coefficients (r) of the standard curves for lipid compound detection; Table S7: Quantitative results of 141 lipid compounds (nmol/g), N/A: Not detected; Table S8: Differential lipid compound information between pileus (Disc) and stipe (St).
