# Supplementary material for: Targeted Metabolomics Resolves Amino Acid and Lipid Specialization Between Pileus and Stipe in Artificially Cultivated Termitomyces upsilocystidiatus
Source: Life (Basel). 2026 May 13;16(5):812. doi: 10.3390/life16050812 (PMC13208876; doi:10.3390/life16050812)
Supplement: Supplementary file 1 [file life-16-00812-s001.zip › life-4260876-supplementary.pdf]

Figure S1. (A) Total ion chromatogram (TIC) for amino acid detection. (B) Overlaid TIC for amino acid detection. (C) Correlation analysis of QC samples for amino acid detection. (D) Distribution of CV in samples from different treatment groups for amino acid detection.
B: High overlap of the total ion chromatograms for metabolite detection, indicating consistent retention time and peak intensity, demonstrates good signal stability of the mass spectrometer when analyzing the same sample at different times. The high stability of the instrument provides important assurance for data repeatability and reliability. C: The diagonal squares represent QC sample names; the lower left off-diagonal squares are scatter plots showing the correlation between the corresponding QC samples, where the X and Y axes represent metabolite content, and each point represents a metabolite; the upper right off-diagonal squares show the correlation coefficients for the corresponding QC samples. D: The X-axis represents the CV value, and the Y-axis represents the proportion of the number of substances with CV values less than the corresponding value relative to the total number of substances. Different colors represent different treatment groups. QC represents quality control samples. The two vertical reference lines correspond to CV values of 0.2 and 0.3. The horizontal reference line corresponds to 80% of the total number of substances.

Figure S2. (A) Total ion chromatogram (TIC) for lipid compound detection. (B) Overlaid TIC for lipid compound detection. (C) Correlation analysis of QC samples for lipid compound detection. (D) Distribution of CV in samples from different treatment groups for lipid compound detection.
B: High overlap of the total ion chromatograms for metabolite detection, indicating consistent retention time and peak intensity, demonstrates good signal stability of the mass spectrometer when analyzing the same sample at different times. The high stability of the instrument provides important assurance for data repeatability and reliability. C: The diagonal squares represent QC sample names; the lower left off-diagonal squares are scatter plots showing the correlation between the corresponding QC samples, where the X and Y axes represent metabolite content, and each point represents a metabolite; the upper right off-diagonal squares show the correlation coefficients for the corresponding QC samples. D: The X-axis represents the CV value, and the Y-axis represents the proportion of the number of substances with CV values less than the corresponding value relative to the total number of substances. Different colors represent different treatment groups. QC represents quality control samples. The two vertical reference lines correspond to CV values of 0.2 and 0.3. The horizontal reference line corresponds to 80% of the total number of substances.
