# Supplementary figures and images for: Targeted Metabolomics Resolves Amino Acid and Lipid Specialization Between Pileus and Stipe in Artificially Cultivated Termitomyces upsilocystidiatus
Source: Life (Basel). 2026 May 13;16(5):812. doi: 10.3390/life16050812 (PMC13208876; doi:10.3390/life16050812)

D

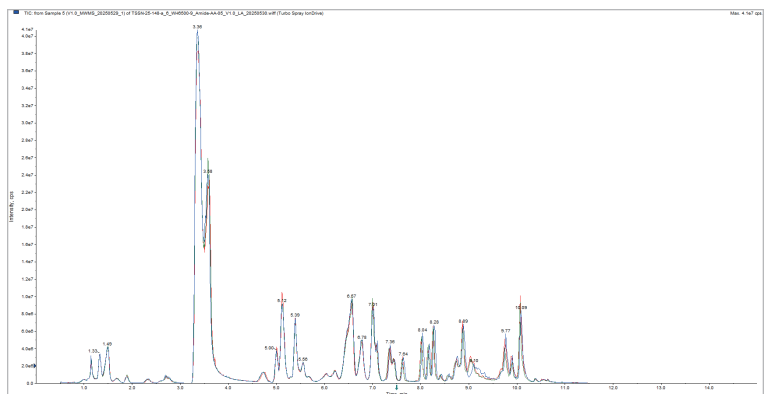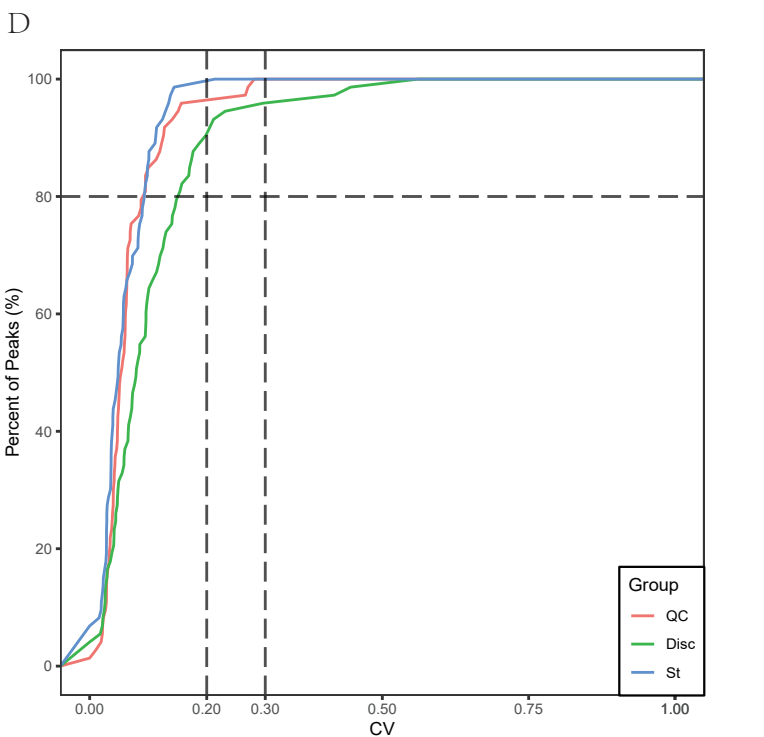

Supplement: Supplementary file 1 [file life-16-00812-s001.zip › Figure S1.pdf]

A

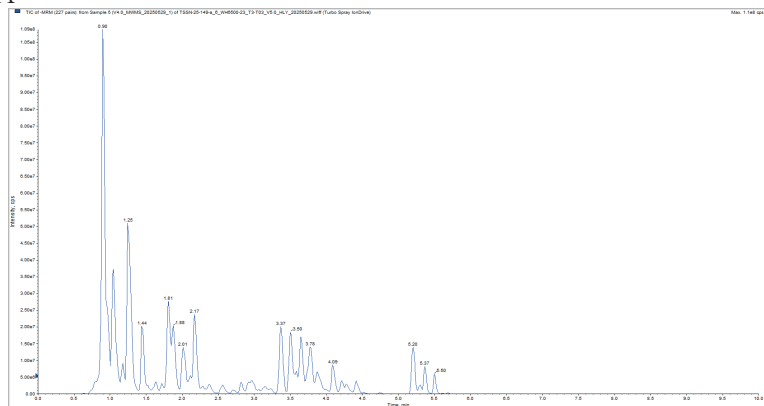

B

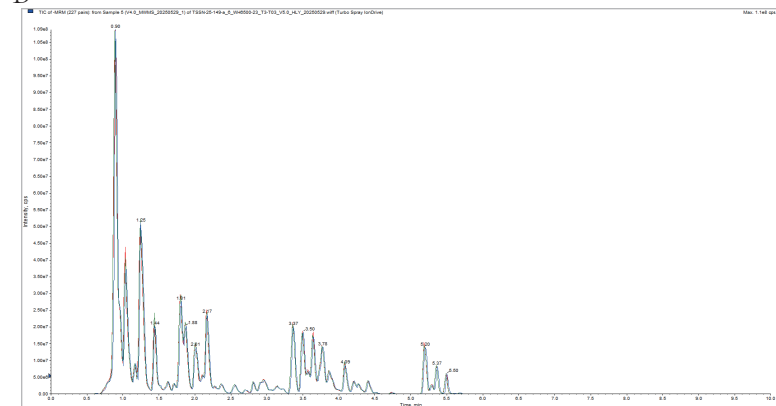

C

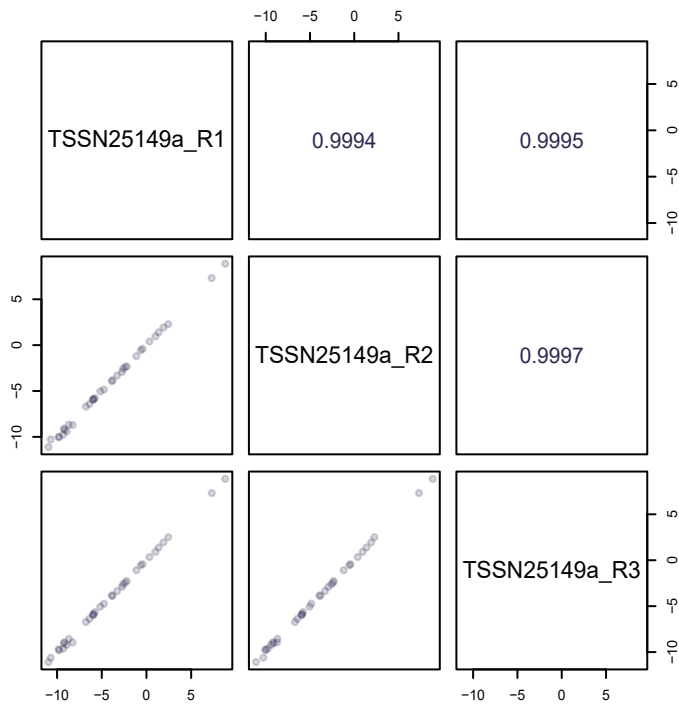

D

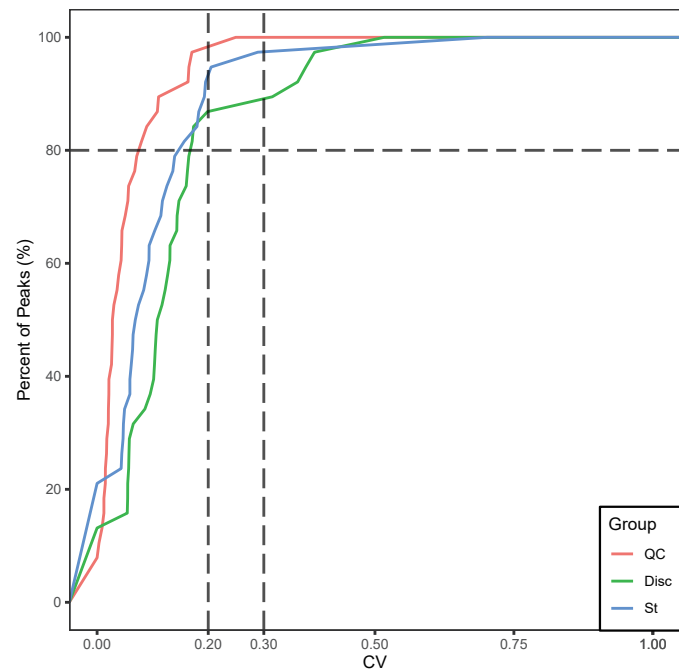

Supplement: Supplementary file 1 [file life-16-00812-s001.zip › Figure S2.pdf]
